# Peritoneal carcinomatosis from a small bowel carcinoid tumour

**DOI:** 10.1186/1477-7819-4-75

**Published:** 2006-11-03

**Authors:** Gonzalo Gutierrez, Ian R Daniels, Ana Garcia, Jose M Ramia

**Affiliations:** 1 Hospital Universitario Virgen de las Nieves, Granada, Spain; 2 Royal Devon & Exeter Hospital, Exeter, UK

## Abstract

**Background:**

Peritoneal carcinomatosis from a gastrointestinal carcinoid tumour is rare and the long-term management and prognosis have not been clearly defined. The natural history is different from gastrointestinal adenocarcinoma, although its capacity to invade regional lymph nodes and generate distal metastasis can make the management more complex. Whilst the development of carcinomatosis is uncommonly reported, it may be higher than expected.

**Case presentation:**

A 63 years-old woman underwent emergency surgery in 1993 for right iliac fossa pain and a mass that was found to be an ileal carcinoid tumour. Over the next ten years, further surgery was required for disseminated disease with peritoneal carcinomatosis and liver metastasis. Systemic chemotherapy had little effect, although Somatostatin was used effectively to relieve symptoms caused by the disseminated disease (flushing and diarrhoea).

**Conclusion:**

Peritoneal carcinomatosis from carcinoid tumours is not well documented in the literature. Aggressive surgery must be performed in order to control the disease since chemotherapy has not been reported to be effective. With repeated surgery long-term survival can be achieved in these patients.

## Background

Intestinal carcinoid tumours can be found in all parts of the gastrointestinal tract, with the highest incidence being in the distal ileum. They present as a spectrum from small 'benign' tumours to the classical 'carcinoid syndrome' of local tumour invasion with liver metastases and systemic symptoms. However, compared to adenocarcinoma, they have a better prognosis and long-term survival is reported. This case reports peritoneal dissemination of the carcinoid tumour and a long follow up of the disease.

## Case presentation

**Figure 1:**
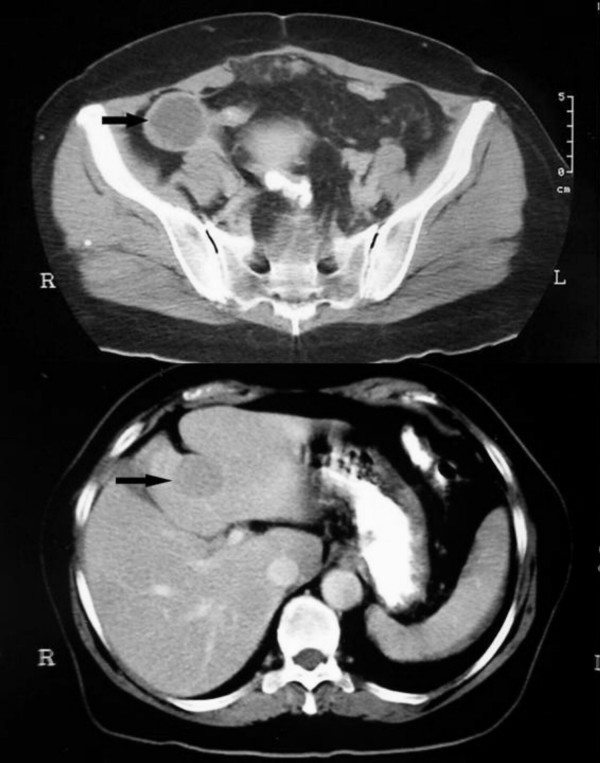
The initial CT scan demonstrates a mass in the right iliac fossa (arrow) and a suspected metasasis in segment IV of the liver.

**Figure 2:**
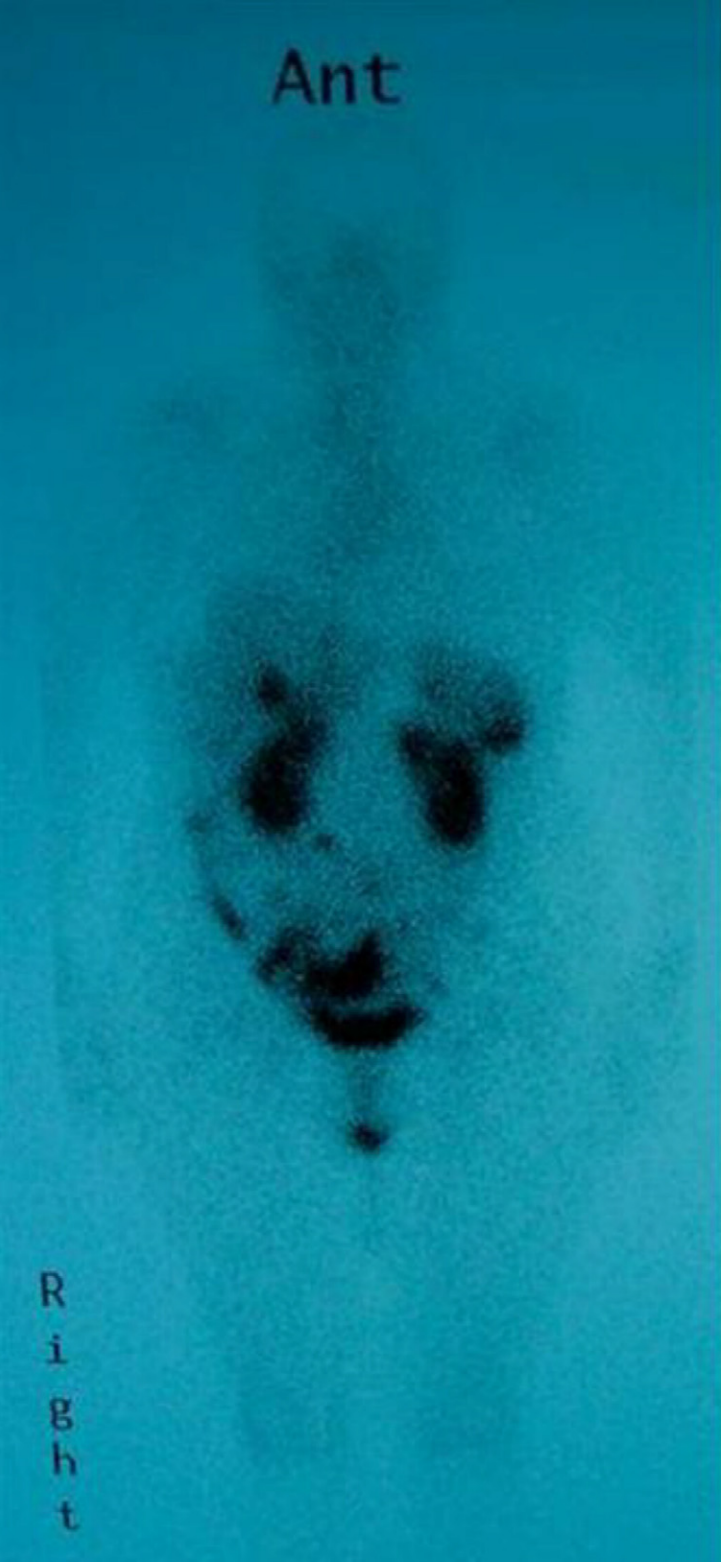
Octreotide scanning of the patient revealed 'hot-spots' in the liver (arrow), ascending colon, the right iliac fossa and upper abdomen.

A 63-year old woman presented in 1993 with a short history of right iliac fossa pain, localised peritonitis, and an elevated white cell count. She reported a two-year history of intermittent colicky abdominal pain. At operation, a 5 cm ileal tumour was found, situated 10 cm proximal from the ileocaecal valve. This was resected. Histopathological assessment confirmed a carcinoid tumour with locally involved nodes (three positive lymph nodes). Nine years later, she re-presented with right-sided abdominal pain, a two-week history of diarrhoea, and a two month history of episodes of her face getting "hot and blushing". A computed tomography (CT) scan revealed a liver nodule (30 mm diameter) and a mass (60 × 40 mm) in the right iliac fossa, which was in contact with a small intestinal loop (Figure 1). Octreotide scanning (indium-111 labelled pentreotide scintigraphy) demonstrated that the liver, the ascending colon, the right iliac fossa, and an area in the superior half abdomen had positive foci (Figure 2). In addition, the tumour marker CA-125 was raised (40.5 U/ml)) and so were the urine levels of 5-hydroxyindoleacetic acid (5-HIAA) (41.2 mg/day). Percutaneous biopsy of the liver lesion confirmed a carcinoid tumour.

At operation in January 2003, peritoneal carcinomatosis was discovered with tumoural implants over the greater omentum, the parietal peritoneum, the left ovary, the terminal ileum (with stenotic and fibrous lesion), and a liver metastasis in segment IV. The peritoneal deposits, left ovary and fallopian tube, the involved distal ileum, and the liver metastasis were surgically removed. All deposits were histologically metastases from the original carcinoid tumour, although the distal ileal tumour metastasis only affected the serosal and muscular layers. Following an uneventful postoperative recovery, she commenced six cycles of chemotherapy with Streptozotocine and 5-fluorouracil.

In October 2004, she was admitted with abdominal cramps and vomiting. A CT scan revealed dilated intestinal loops that appeared to be attached to each other and with the anterior abdominal wall. In view of the previous surgery and the history of carcinomatosis, she underwent a further laparotomy. However, at operation, the cause was adhesional obstruction, although some foci were found in the parietal peritoneum and in the Pouch of Douglas. These implants were less than 4 mm in length and were excised. All the biopsies were positive for carcinoid tumour and after an uneventful recovery she was discharged. Further therapy with Somatostatin with the aim to control the progression of the disease and because she reported occasional flushing, achieved a good quality of life. At review in March of 2006, the patient reported some abdominal pain and infrequent flushing, but is otherwise well. A further Octreotide scan revealed no disease activity.

## Discussion

The first reported case of a carcinoid tumour was published in 1888, but the molecular basis of the syndrome was not elucidated until 1954 and the following year the presence of serotonin was confirmed [1]. The serotonin secreting cells have particular histological features (positive reactions to silver stains, enolase, synaptophysin, and chromogranin), and arise from the enterocrhomaffin or Kulchitsky cells [2].

There is no globally accepted classification for carcinoid tumours although most series and reports are based upon the embryologic origin of the organ and its blood supply. Hence, they can be divided into; foregut (lungs, oesophagus, stomach, liver and upper duodenum), midgut (small intestine and proximal colon), and hindgut (distal colon and rectum) carcinoid tumours [1-4]. Depending on their origin, they secrete different hormones and substances. Such reported examples include; bombesin, growth hormone, adrenocorticotropin hormone, glucagons, catecholamines, gastrin, insulin, and serotonin[1,4]. However, serotonin secretion is usually associated with midgut carcinoids [5].

The incidence of carcinoid tumours is reported to be 0.5 – 8.4 cases per 100,000, although they have been reported to be present in 1% of autopsies [4,6,7]. The principle site is the gastrointestinal tract, followed by the lung and bronchus [7]. The appendix was the most frequent site within the GI tract, followed by the rectum and the ileum. However, more recent data from the National Cancer Institute Surveillance, Epidemiology, and End Results (SEER) program (1973–1997) has quantified the prevalence of carcinoid in the different sites, with the majority of carcinoid tumours occurring in the gastrointestinal tract (54.5%), followed by the lung and bronchus (30%), pancreas (2.3%), gynaecological/ovarian (1.2%), and the biliary tract (1.1%). Within the gastrointestinal tract, the small intestine (44.7%) is the most frequent site, followed by the rectum (19.6%), appendix (16.7%), colon (10.6%), and the stomach (7.2%) [8,9]. Within the small intestine, the ileum is the principal site of origin, occurring in greater than fifty percent of cases.

The symptoms of intestinal carcinoid tumour are non-specific and vague, generally consisting of periodical abdominal pain as a major symptom, along with intestinal obstruction, intermittent diarrhoea, and gastrointestinal bleeding [1,5,10]. Intestinal obstruction, due to a desmoplastic reaction from the tumour causing local mesenteric fibrosis, can lead to the need of surgery and to the incidental finding of a carcinoid tumour [6]. The incidence of carcinoid syndrome is variable, reported to occur in 1.6–18% of cases, depending on the location of the primary tumour, local tumour extension, and the secretion of serotonin and other amines [2,5,10,11]. Therefore, ileal carcinoids are more likely to develop the syndrome with almost 90% of patients with carcinoid syndrome having a small bowel carcinoid [5]. Whilst the development of hepatic metastasis has been a common established prerequisite for the development of carcinoid syndrome, it is not essential [2,11]. Despite the fact that serotonin is almost always present in carcinoid syndrome, the classical symptoms of the syndrome, flushing, diarrhoea, intestinal cramps, heart valves lesions, asthma and wheezing are thought to be caused by other amines, with one example being flushing caused by bradykinin [2,11]. Nevertheless, as many as 40–60% of patients are asymptomatic and as the symptoms are often non-specific, this may lead to a delay in diagnosis with a range from 2–20 years. In one series, the median delay for abdominal carcinoid tumours was 5.5 years, with a maximum delay of 22 years, although 12% had no delay [10]. At diagnosis, 10% had only a primary tumour, 20% had lymph node metastases, 41% liver metastases, 16% carcinomatosis, and 13% had extra-abdominal metastases. These findings correlate with other studies [6,11].

Carcinoids are usually discovered during laparotomy for intestinal obstruction or pain, and the existence of multiple primary tumours is remarkable in 26–28% of cases [5,12]. The evidence suggests that this multiplicity is generated by metastases from the primary tumour [13]. They are more aggressive than other intestinal carcinoids, with transmural invasion (77–78%), lymph node metastasis (31–66%), and liver or mesenteric metastasis (10–32%) [5,11,12]. This is associated with a poorer prognosis, with adjusted five-year of 58%, as compared with 86–92% for appendiceal tumours [9,11]. The five-year overall survival for all carcinoid tumours varies from 70–75% for localized tumours [2,8].

With hepatic metastasis, the ten-year survival is reported to be 20%, however, recent study shows 5-year survival between the groups with non-aggressive and aggressive liver resection to be 25% versus 72% [6,14]. The median survival in patients with the carcinoid syndrome is 3.5–8.5 years [5].

Peritoneal carcinomatosis from carcinoid tumours has been scarcely reported in the form of a limited number of case reports. Recently, in a series of 116 cases of intestinal neuroendocrine tumours [15], thirty were carcinoids. Within this series, the prevalence of peritoneal carcinomatosis was 27% (8/30). Ileal carcinoid tumours were the most frequent source of peritoneal carcinomatosis in 7/8 patients, and 6/8 had synchronous liver metastasis. The five-year survival for carcinoid tumours with and without peritoneal carcinomatosis was 65% versus 75%, although no deaths were related to the carcinomatosis. For all the intestinal neuroendocrine tumours within this series, the five-year survival with or without liver metastasis was 74% and 94 %, respectively. Again, the interval between diagnoses from primary tumour to peritoneal carcinomatosis was very long (overall median time 20 months, 0–273 months), and from symptoms to diagnosis was 54 months. Life expectancy was not affected by the carcinomatosis, but it was if liver metastases were present.

In another large series of 111 endocrine carcinomas that were surgically treated [16], 81% (30/37) were carcinoids (the vast majority from the ileum, 83% (25/30)), with peritoneal carcinomatosis identified in (37/111). In this series, 17 patients with peritoneal carcinomatosis underwent radical surgery plus intra-peritoneal chemotherapy, with the procedures being described as palliative, but with the aim of reducing bowel obstruction and pain. They had no postoperative mortality, and had a morbidity rate of 47%. Five year survival was 40.9% for those in whom complete resection was not possible, (cause of death being from liver failure in 60% and bowel obstruction in 40%) and 66.9% for those with cytoreductive surgery plus intraperitoneal chemotherapy (deaths from liver failure 23.5%, bowel obstruction 5.8% and cerebral haemorrhage 5.8%. They conclude that carcinoid carcinomatosis is direct cause of death in 40% of cases if no specific therapy is used.

In our particular reported case of peritoneal carcinomatosis from a carcinoid tumour, ten years passed from the initial surgery to the carcinomatosis, although the patient related colic abdominal pain two years before the emergency operation. This was probably due to the ileal carcinoid tumour. Whilst colic is generally the principle symptom, it was vague and did not lead to investigation of a possible cause, and is likely the reason for the delay in diagnosis. Radical surgery is the only potential curative therapy and Somatostatin or analogues have been widely used as good effectors for control of symptoms [4,5,17] The anti-proliferate potential of Somatostatin and analogues, even for reduction of metastasis, as indicated elsewhere [17-19], is questionable and further evaluation is warranted. The use of radio-labelled Somatostatin analogues for advance carcinoids has been previously published [18]. Indium-DTPA-octreotide has a low response rate (around 10–20%), yttrium-DOTA-Tyr3-octreotide has reported rates of complete remission in the 20–30% range, and a more recently developed compound, lutetium-DOTA-Tyr3-octreotate, has a reported rate of complete remission in approximately 40% of cases [19,20]. However, further investigation is needed before a consensus can be reached. Furthermore, other drugs such as 5-fluorouracil, streptozotocin, doxorubicin, leucovorin, and interferon have been employed with different combinations, but the responses have been poor [5]. The use of intraperitoneal chemotherapy has little evidence, but the progressive use of this method associated with extensive cytoreductive surgery for other types of tumours (carcinomatosis from colon adenocarcinoma, ovarian adenocarcinoma and appendiceal mucinous cystadenoma) make this a suitable and promising technique.

## Conclusion

Radical surgery, aimed at local cure with ileal carcinoids, is associated with a better prognosis and longer survival. Improvements in quality of life can be achieved using Somatostatin, but progression of the disease is predictable. The surgeon can approach these tumours with the knowledge that carcinomatosis is more common than expected. Survival is longer compared with adenocarcinoma and therefore maximal efforts must be achieved to control the disease.

## Conflict of interest

The author(s) declare that they have no competing interests.

## Authors' contributions

**GG: **Documented and prepared the draft.

**IRD: **Revised, edited most of the manuscript and helped in preparing the draft.

**AG: **Surgeon who performed the operations and helped in preparing the manuscript specially in describe the findings and the follow-up.

**JMR**: Literature search, revision of bibliography and helped with the editing of the manuscript.

All authors read and approved the final manuscript for publication
